# Hopes and fears: A qualitative analysis of the intern perspective at the start of EM residency

**DOI:** 10.1002/aet2.10764

**Published:** 2022-06-23

**Authors:** Korie Zink, Cory Clugston, Linda Regan

**Affiliations:** ^1^ Department of Emergency Medicine Johns Hopkins University School of Medicine Baltimore Maryland USA

## Abstract

**Objectives:**

Most emergency medicine (EM) residency programs have orientation curricula to guide interns through the transition from medical school to residency, although no standard components are required. This transition is recognized as a challenging time for young physician learners; however, there is no current understanding of the perspective of incoming interns as they enter residency. We sought to identify themes, examine the current literature, and reflect on the experiences of our residency leadership to inform the creation of orientation activities that foster positive experiences, as well as directly address intern fears and anxieties.

**Methods:**

This qualitative study collected free text responses on the first day of EM orientation regarding areas of high excitement and high fear as interns entered residency. Data were collected from 2011 to 2019 in a 4‐year EM residency program and a 6‐year combined EM‐Anesthesia residency program in the Mid‐Atlantic. An inductive approach was used to code intern responses and develop themes within each category, and a frequency analysis was performed.

**Results:**

A total of 112 interns participated. Thematic analysis of coded responses yielded 11 themes around “most excited” and 13 themes around “most scared.” The most frequent themes for “most excited” were: (1) Forming new relationships, (2) Building and applying knowledge, and (3) Being responsible for the care and education of others. For “most scared,” the most frequent themes were: (1) work–life balance and burnout, (2) making harmful mistakes, and (3) knowledge wealth and retention.

**Conclusions:**

We identified themes of high excitement and high fear for interns entering residency orientation. Based on the themes developed and current literature, recommendations for residency programs regarding intern orientation are provided, specifically that programs intentionally address opportunities for professional identity formation, building relationships with new people/places, emphasizing wellness, and mitigating burnout.

## INTRODUCTION

Emergency medicine (EM) is a field with a broad clinical scope, requiring students to enter residency with knowledge and skills curated from varied exposures across multiple disciplines in medical school. Historically, most EM residency programs have designed orientation programs to facilitate the transition for their incoming interns into residency.^1^, ^2^ However, there is no standardized orientation curriculum for EM interns in the United States.

Much of the existing literature on the transition from medical school to residency explores “boot camp” courses offered at the end of medical school to reinforce knowledge and build skills needed to begin residency,^3^, ^4^ with the bulk of the existing literature on residency orientation curricula also focusing on medical knowledge and skill‐based content.^2^, ^5^, ^6^ Beyond the acquisition of knowledge and skills, there are numerous stressors inherent to this transition, with data to suggest that resident health and well‐being may decline throughout intern year.^7^, ^8^, ^9^ Additionally, medical students transitioning to residency come from various backgrounds and have inherent differences in knowledge, skills, and attitudes.^5^, ^10^

Despite this critical time of transition, published data on orientation programs have had little to no focus on the perspective of interns entering EM residency.^1^, ^2^ While the perspective of educators is clearly an important element in designing a robust and meaningful orientation program, the learners involved in those programs are key stakeholders that have been overlooked. This qualitative study aimed to: (1) explore the perspective of interns entering EM residency, focusing on the aspects of residency interns were most excited and most scared about as they transitioned from medical school to post‐graduate training and (2) create curricular recommendations for residency program directors in EM.

## MATERIALS AND METHODS

We followed an inductive approach to code intern responses looking at areas of high excitement and high fear during intern orientation. Using a constructivist paradigm,^11^ we then conducted a thematic analysis to generate key themes. To ensure we accurately represented the scope of our data, we also conducted a frequency analysis by mapping individual coded responses to our global themes.

### Study setting and population

The study took place in an emergency medicine residency at an urban academic center with a 4‐year EM residency program and a 6‐year combined EM‐Anesthesiology residency program in the Mid‐Atlantic. Interns from both the EM and combined EM‐Anesthesiology residencies complete the same intern year and orientation program. All interns who took part in our standard orientation program were eligible to participate from 2011 through 2019, with data collection intentionally stopped with the first cohort completing their intern orientation during the COVID‐19 pandemic. No intern during the recruitment timeframe was excluded or declined to participate.

### Data collection

During the first day of orientation, and before any activities began, all interns were provided with a blank index card by the program director. They were instructed to “write down the top items you are the most excited about as you start residency on the front and the top items you are the most scared about as you start residency on the back.” There was no item limit provided and no suggestions for items to consider were provided. Interns were instructed to not write their names on the cards and informed that they would be read back anonymously to the group in a future session. Collection took less than 5 min for all groups. Cards were collected and stored in a locked cabinet in the residency office until the future session when they were read back to the group without attribution and used as lead points for discussion. They were then returned and kept in the locked cabinet until data analysis. This process was repeated annually over 9 years during intern orientation from July of 2011 through July of 2019. Once all data were collected, it was transcribed into two sheets by the principal investigator (PI) using Microsoft Excel Online^Ⓡ^ and stored securely in Microsoft OneDrive (Microsoft Office OnlineⓇ).

### Data analysis

To build a shared experience and understanding, all three investigators coded the first year of intern data (51 scared responses that yielded 30 codes and 52 excited responses that yielded 26 codes). Multiple codes could be assigned to a single response, if the intended meaning was felt to require this delineation. For example, one response to the Excited prompt was “knowing how to do things and actually save lives.” This was assigned two codes: “knowing what to do” and “making an impact/helping people.” All subsequent responses were coded independently, in a blinded fashion, by two study co‐investigators. After initial review and coding, the PI unblinded assigned codes for group review; a third investigator adjudicated coding discrepancies. Codes were added to a master codebook that was kept by the PI. Codes were merged, expanded, and collapsed with each subsequent year of trainee data. Thematic analysis of the codes yielded 11 themes around high excitement and 13 themes around high fear.

**FIGURE 1:**
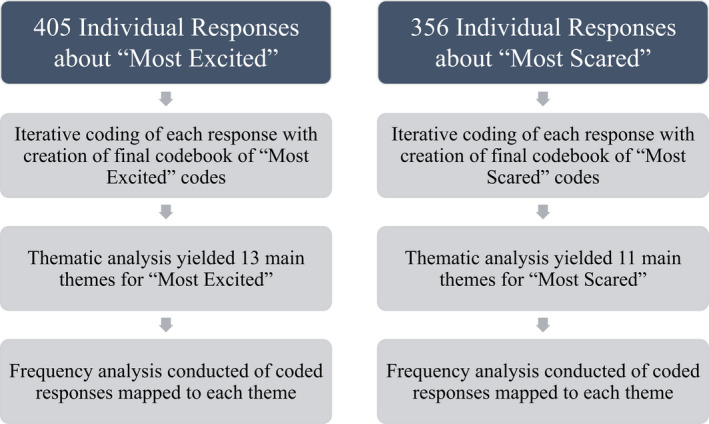
Flow diagram of coding process

In addition to the thematic analysis, a frequency analysis was also conducted, whereby each coded response was counted and mapped to the theme it had informed. See Figure 1.

The IRB deemed this study exempt research.

### Reflexivity

We acknowledged the potential impact of our past experiences during data analysis in this constructivist paradigm. All three authors are emergency physicians. The PI is the current residency program director, and the other two co‐investigators are medical education fellows. We reflected on this potential influence at the start of the study and throughout data analysis in order to minimize bias.

## RESULTS

A total of 112 EM interns completed the activity on day one of intern orientation yielding 405 individual responses of “most excited” items and 356 individual responses of “most scared” items. One item was unable to be deciphered and was excluded from analysis, yielding 355 individual statements for coding of “most scared” items. These codes were analyzed, and themes were generated yielding 11 themes around “most excited” and 13 themes around “most scared.” These themes are presented in Tables 1 and 2.

**TABLE 1:** Themes identified that interns are the most excited about

Excited Themes	Description	Percentage of responses mapped to theme (%)
Forming new relationships	The building of both work and personal relationships with new colleagues and mentors as well as the patient care team	21.20
Building and applying knowledge	Referencing both pure knowledge acquisition as well as applying the knowledge (e.g.^a^ knowing what to do and when to do it)	14.90
Being responsible for the care and education of others	The increase in responsibility associated with patient care as well as the impact one can have on patients and other learners	13.70
New city	Getting to be in and explore a new city as well as its local cuisine and culture	10.00
Identity as a “physician”	Being a “doctor” and its associated identity (e.g., wearing a white coat)	9.40
Adulthood	Experiencing the benefits of independent adult life and starting a new phase in life (e.g., making money, moving in with a partner, having personal responsibilities)	8.20
Having a clinical/EM focus	Being able to focus on EM^b^ as their primary specialty as well as build a niche within the field of EM (e.g. ultrasound, toxicology)	7.30
Goals and personal growth	Achieving short‐ and long‐term personal goals as well as personal growth	5.30
Opportunities specific to training program	Being able to take advantage of both the reputation of the program as well as the unique resources associated with the program and institution	5.30
Being done with medical school	Being able to return to the clinical area and in general, not be a student anymore	2.50
Increasing autonomy	Increased independence and expectations for autonomous decision‐making (e.g., writing prescriptions)	2.20

^a^ e.g., for example.

^b^ EM, emergency medicine.

**TABLE 2:** Themes identified that interns are the most scared about

Scared Themes	Description	Percentage of responses mapped to theme (%)
Work–life balance and burnout	Not being able to balance demands of work and personal life, leading to burnout (e.g.,^a^ emotional exhaustion, depersonalization)	17.60
Making harmful mistakes	Making medical errors that cause harm or death to a patient	14.60
Knowledge wealth and retention	Lacking medical knowledge or not remembering things that they learned in medical school	13.90
Being responsible for the care and education of others	The increase in responsibility associated with patient care as well as the impact one can have on patients and other learners	11.70
Feelings of inadequacy or embarrassment	Feeling inadequate compared to peers (e.g., imposter syndrome), worry over being involved in embarrassing situations	9.50
Specific EM^b^ skills and populations	EM‐specific skills, such as high acuity clinical scenarios or fine motor skills (e.g., codes, needle procedures) and populations such a pediatric patients, pregnant patients	9.00
Logistical adaptations	Adjusting to a new environment, such as a large hospital, electronic medical record	5.40
Not fitting into local culture	Not fitting into the new community or making friends or feeling that your personality does not mesh with your peers	4.70
Goals and achievement	Not living up to personal or family goals/expectations	4.50
New city/life transition	Adjusting to a new living situation, new city, or other new situations in a big life transition	3.60
Physical illness/injury/safety	Experiencing an occupational injury or illness, as well as workplace violence	2.90
Increasing autonomy	Increased independence and expectations for autonomous decision‐making (e.g., writing prescriptions)	2.00
Negative interpersonal interactions	Experiencing confrontations with coworkers or patients	<1

^b^ EM, emergency medicine.

^a^ e.g., for example.

Of the interns that participated, 71 self‐identified as male (63.4%) and 41 self‐identified as female (36.6%); 64 self‐identified as white or Caucasian (57.1%), 20 as from Asian descent (17.9%), 17 as black or African American (15.2%), 5 as Hispanic (4.5%), 2 as Indian (1.8%), and 2 as Lebanese (1.8%).

## DISCUSSION

We found commonality amongst the areas of excitement and fear across 9 years of EM interns starting residency. Program directors in EM may find benefit from reviewing the list of our themes and determining which are optimal for intervention based on their own trainees, local environment, and available resources. While some areas were anticipated by our team, such as the focus on knowledge building, we found areas that represent a unique trainee perspective that can be used to inform future orientation programs. We highlight four such areas below, chosen given the relative frequency of their report, as well as the opportunity they offer for targeted intervention.

### People and places

The path to becoming a physician involves multiple transitions en route to independent clinical practice, with the transition from medical school to residency being both exciting and particularly challenging.^10^, ^12^, ^13^, ^14^, ^15^ Not unexpectedly, the most common theme for high excitement reported by our cohort centered around forming new relationships (21%), with an additional 10% of respondents reporting excitement about their new city or local culture. Programs should capitalize on these themes by incorporating activities and social events focused on relationship building for peers, mentors, and colleagues such as nursing, pharmacy, or other staff that function as part of the patient care team in the ED.

Activities that familiarize the interns to their new city can help residents from both a practical and a leisure standpoint (e.g. learning about transportation, food), as well as address the small percent of our residents that may be intimidated by being in a new place, as was seen with our respondents. Lastly, these types of activities can also serve to expose interns to the history of their new city and the culture of their patients. For example, at our program, we take our interns into the community on a tour of local murals to both learn about local neighborhoods as well as spark greater curiosity about the communities and culture of the patients we serve.^16^

### Medical knowledge

Knowledge was a high frequency item on both lists. Interns were excited about building and applying medical knowledge, while also anxious about whether their wealth of knowledge was adequate and if they could retain it. The majority of published intern orientation curricula focus on the building and expansion of foundational knowledge and procedural skills, as well as guarding against knowledge degradation between medical school and intern year.^1^, ^2^ While programs can consider baseline knowledge assessments and early development plans, interns’ fear of not having enough knowledge at the start of residency could also reflect a lack of comfort with certain learning skills. Compared to the structured learning environment of medical school, learning in the less controlled environment of residency training requires a different set of learning skills.^14^ Intern orientation remains an important opportunity for practice with experiential and skill‐based learning and interns may benefit from specific skill development on learning how best “to learn” in this new environment.^17^

Chang et al. found that medical students transitioning to residency preferred and benefited from an immersive introduction to clinical training, which gradually exposes them to real‐life environments and the skills and knowledge needed to succeed clinically.^14^ Based on these findings, in our program we employ intern “shadow shifts,” in which interns are able to observe a current resident during a real clinical shift and participate in clinical decision making in a low‐stakes, highly supportive setting. As orientation progresses, the shadowing experience can transition into “buddy shifts,” in which interns work actual clinical shifts, but in pairs, such that they have early exposure to the authentic clinical environment while benefiting from the additional support of a peer.

### Professional identity formation

Chang et al. found that a key component of the transition from medical school to residency is the process of professional identity formation, which was an area of high excitement for our interns.^14^ Nearly 10% of our respondents indicated that their new identity as “physician” was exciting to them, and even more (14%) were excited about the new responsibility of caring for and educating others, which is inherent in the role of doctors. This responsibility was also a source of high fear for 11% of interns. Despite most orientation programs describing a focus on clinical skills and medical knowledge, administrative skills specific to departmental needs, and clinical competency assessment for core skills, such as procedures,^2^, ^18^ none of the programs described how they addressed the need to socialize interns into their new profession and support their new identities. These identities, complete with high stakes responsibilities, created both positive and negative emotions for our group. The literature suggests that interns struggle with professional identity formation and that educators tend to underestimate the impact of this struggle on their transition to residency.^10^, ^19^ Coaching may be beneficial to building professional identity during this transition, particularly when guided by faculty experienced in the process of reflection, integrating personal experiences, and openly discussing learner fears.^14^, ^20^

Though there are potential benefits to focusing on professional identity formation during intern orientation, Radcliff and Lester highlight the potential dangers of socialization to traditions that are long‐standing in medicine, such as “learning by embarrassment.”^14^, ^15^ Programs should take the opportunity to explicitly discuss the benefits and pitfalls of this new professional identity, including the “hidden curriculum” in medicine^21^, ^22^ and its possible negative impact on professional identity. While programs must continue to work with their faculty to establish expectations for how the learning environment can best support a healthy learning experience, interns should be encouraged to raise concerns about any experiences they feel have been inappropriate, derogatory, or embarrassing and instructed on how to report them. Additional topics to consider including to help build a healthy professional identity include how to respond to microaggressions and how to advocate for themselves and others in vulnerable positions. We recommend emphasizing a zero‐tolerance policy for the historically tacit practices that lead to negative emotions in learners (e.g., “pimping,” public humiliation) in the hope that this will diminish the likelihood of the negative effects of the hidden curriculum being integrated into a developing physician’s identity formation.

### Emotional wellness/burnout

The theme that inspired the most anxiety for our intern cohort was “Work–life balance and burnout.” In general, this has become an increasingly prominent theme in medical training, and with good reason—current data suggest that interns experience significant decline in overall wellness, with the risk of depression and suicide climbing during the early months of residency.^7^, ^9^, ^23^ The fact that interns are arriving to residency with this as their most frequently reported fear suggests that they are keenly aware of the declining wellness of their peers and mentors.

Growing rates of physician suicide have led to national recommendations to promote mental health amongst trainees, which include education about burnout and emotional distress.^24^ One unique approach to addressing burnout in interns suggested in the literature is involving resident families in orientation and educating them on the signs of burnout.^25^ Through education on burnout and the struggles inherent to residency training, family members may be more equipped to recognize early signs of burnout and support their loved ones in a meaningful way.^25^ No matter what approach programs choose to take, intern anxiety related to burnout and emotional wellness should be addressed through education about recognizing burnout in themselves and others and the importance of self‐care. Programs should educate interns about the mental health resources available to them and destigmatize the use of these resources. One way we have found to destigmatize the use of mental health resources is to directly introduce them during intern orientation; our program has each intern meet one on one with a mental health counselor during orientation to familiarize them with the availability of and process for reaching out to these services.

The second most prevalent fear was “Making harmful mistakes.” Most students who prepare to enter residency have a history of high academic achievement. Given the challenges of residency, these perpetual high achievers will inevitably experience failures in their training, leading many into uncharted territory. Making harmful mistakes and burnout are closely linked.^26^, ^27^ Perceiving that one has caused harm to others through a mistake, with the resultant shame many may experience in this setting, can have a significant impact on emotional and physical health. In 2008, White et al. noted that 98% of medical and surgical residents report having been involved in medical errors with 45% reporting that the error was serious.^28^ Despite the frequency of involvement in medical errors, only 33% of residents had received training in error disclosure.^28^ Physicians who had experienced a more serious error endured more significant negative mental health effects including anxiety over future errors, loss of confidence as a physician, ability to sleep, and job satisfaction.^29^

Fatima et al. suggest incorporating training regarding disclosure, discussion, and coping responses to medical error into the standard residency curriculum.^27^ As the culture of medicine continues to evolve to support the normalization of admitting deficits and recognizing the value of struggle in the acquisition of new knowledge and skills, programs should heavily emphasize during intern orientation that failure and growth are normal during residency.^30^ We believe this will help to quiet some of the fear of making mistakes and the associated feelings of inadequacy or embarrassment. The combination of the substantial negative impact on physicians involved in a significant medical error and the frequency with which they are involved in these errors highlights this topic as essential to address at this critical time of transition to residency.

## LIMITATIONS

This study represents a single cohort of EM resident opinions at a single institution and may not be generalizable to a larger, national resident population. While this is a single site study, the cohort of incoming interns represented medical schools from all over the country whose data were collected prior to any exposure to local orientation content, making the findings more generalizable than a typical single site study. One strength of our data comes from the size of our cohort. In contrast, the cohort is composed of interns in aggregate spanning over 9 years and may not reflect changes in fears or excitement as they changed over time, due to changes in the landscape of medicine. We intentionally chose to end our cohort in 2019 and not include any data after the COVID‐19 pandemic started, given the impact we believed this may have had on the areas interns were most scared about, While it is possible that our data may not capture the concerns of a current intern arriving to an EM program while the pandemic is still an active part of day‐to‐day life, we believe that these themes are indeed applicable across incoming interns and the curricular interventions we suggest will benefit new members of any emergency medicine department.

Similar to any qualitative work, the authors’ own personal biases may have impacted how we interpreted and coded the meaning of statements. We believe, however, that our collective time working in education leadership, as well as having just completed residency training, give our team a unique collective perspective on the data. Lastly, our understanding of what is currently implemented in EM orientation curricula is limited by what has been published in the literature and may not accurately reflect what is occurring in programs around the country.

## CONCLUSIONS

Interns entering emergency medicine residency have common areas of excitement and fear that can be used to inform the development of robust and impactful orientation programming. While the development and retention of medical knowledge is important to interns as they transition from medical school to residency, interns are most excited about meeting new people and building new relationships and most scared about work–life balance and burnout. We recommend that programs review their orientation curricula with a more intentional focus on opportunities such as relationship building with peers and mentors, exposure to their new community and culture, professional identity formation, and the promotion of self‐care and wellness.

## AUTHOR CONTRIBUTIONS

LR was responsible for the study concept and design and acquisition of the data. KZ, CC, and LR analyzed and interpreted the data. KZ, CC, and LR drafted the manuscript. KZ, CC, and LR were responsible for critical revision of the manuscript for important intellectual content. Statistical expertise was by KZ, CC, and LR. KZ, CC, and LR provided administrative, technical, and material support. LR was responsible for study supervision.

## CONFLICTS OF INTEREST

KZ, CC, and LR report no relevant conflicts of interest.
